# A Hardware-Friendly Low-Bit Power-of-Two Quantization Method for CNNs and Its FPGA Implementation

**DOI:** 10.3390/s22176618

**Published:** 2022-09-01

**Authors:** Xuefu Sui, Qunbo Lv, Yang Bai, Baoyu Zhu, Liangjie Zhi, Yuanbo Yang, Zheng Tan

**Affiliations:** 1 Aerospace Information Research Institute, Chinese Academy of Sciences, No. 9 Dengzhuang South Road, Haidian District, Beijing 100094, China; 2 School of Optoelectronics, University of Chinese of Academy Sciences, No. 19(A) Yuquan Road, Shijingshan District, Beijing 100049, China; 3 Department of Key Laboratory of Computational Optical Imagine Technology, CAS, No. 9 Dengzhuang South Road, Haidian District, Beijing 100094, China

**Keywords:** convolutional neural networks, hardware-friendly, low-bit quantization, shift operation, hardware resource occupancy

## Abstract

To address the problems of convolutional neural networks (CNNs) consuming more hardware resources (such as DSPs and RAMs on FPGAs) and their accuracy, efficiency, and resources being difficult to balance, meaning they cannot meet the requirements of industrial applications, we proposed an innovative low-bit power-of-two quantization method: the global sign-based network quantization (GSNQ). This method involves designing different quantization ranges according to the sign of the weights, which can provide a larger quantization-value range. Combined with the fine-grained and multi-scale global retraining method proposed in this paper, the accuracy loss of low-bit quantization can be effectively reduced. We also proposed a novel convolutional algorithm using shift operations to replace multiplication to help to deploy the GSNQ quantized models on FPGAs. Quantization comparison experiments performed on LeNet-5, AlexNet, VGG-Net, ResNet, and GoogLeNet showed that GSNQ has higher accuracy than most existing methods and achieves “lossless” quantization (i.e., the accuracy of the quantized CNN model is higher than the baseline) at low-bit quantization in most cases. FPGA comparison experiments showed that our convolutional algorithm does not occupy on-chip DSPs, and it also has a low comprehensive occupancy in terms of on-chip LUTs and FFs, which can effectively improve the computational parallelism, and this proves that GSNQ has good hardware-adaptation capability. This study provides theoretical and experimental support for the industrial application of CNNs.

## 1. Introduction

In recent years, CNNs have played an important role in various computer-vision techniques, such as image classification [1,2,3], target detection [4,5,6,7,8], and semantic segmentation [9,10]. As the research on CNNs progresses, the scale of CNN models becomes deeper and wider, while increasingly high accuracy is obtained, which leads to an inevitable increase in the amount of computation and parameters in the models. For example, the original VGG-16 model has more than 130 million parameters, has more than 500 MB of memory storage space, and requires more than 30 billion floating-point operations to complete an image-recognition task [1]. This requires hardware devices with more computing and storage resources as support. In addition, some industrial application areas, such as intelligent cars, unmanned aerial vehicles, and radars, which most often use CNNs for real-time data detection and processing, have the same requirement for the deployment of CNN models in embedded devices: high efficiency, high real-time accuracy, and low energy consumption [11,12,13,14]. However, embedded devices, such as FPGAs, often provide relatively few computation and storage hardware resources due to their limited size and energy consumption [15]. Therefore, when CNN models are deployed to FPGA platforms, they often need to reduce computational parallelism or crop parameters to keep devices running properly [16,17]. These operations make it difficult to achieve the balance of efficiency, accuracy, and overhead required in industrial applications.

In order to solve the above problems, it is usually necessary to design software and hardware co-optimization in terms of both the compression of CNN models in software development and the improvement of the hardware friendliness of the models from a hardware-design perspective to improve the computational efficiency of CNNs on FPGA platforms. Currently, the main solutions for CNN model compression are model pruning [18,19] and parameter quantization [20,21]. Parameter quantization is an important and commonly used method, which refers to the sharing [22,23,24] or streamlining [25] of model parameters, such as weights and activation, without changing the model structure. Several studies [26,27] have demonstrated that quantizing the 32-bit floating-point weights in CNN models to 8-bit is sufficient to ensure the accuracy of CNN models without decline. However, the hardware-friendly features invite us to consider both the CNN model’s size and its computational complexity. First, we need to quantize 32-bit floating-point numbers into lower-bit fixed-point numbers to reduce storage-resource usage and improve memory-access efficiency. Second, some previous works [28,29,30] deployed YOLO v2, MobileNet, and VGG-16 models on the ZYNQ XC7Z045 and XC7Z100 platforms; the on-chip DSP resource occupancies reached 88.9%, 95.3%, and 86.7%, while the LUT resource occupancies were all in the range of 50%. These two ZYNQ chips are already higher-end products in the FPGA family with relatively rich on-chip hardware resources. However, the DSP resources on them are almost completely consumed, which not only tends to lead to layout and routing failure of the system and an increase in the difficulty of the work, but it also indirectly proves that DSP resources are the primary constraint on the achievement of high-computational-efficiency CNN models in low-end and mid-end FPGAs. Furthermore, the paper [31] mentions that the actual computational efficiency of on-chip DSPs is about 1/4 of that of other on-chip logic units. Therefore, it is necessary to use simple logic operations (AND, OR, shift, etc.) instead of complex operations (multiplication, etc.) in the hardware to reduce the use of DSP resources [32].

Combining the above two points, Courbariaux et al. [20] and Rastegari et al. [21] proposed a binary weight approximation neural network and XNOR-Net, which quantize all the weights in CNNs to −1 and +1. Subsequently, the ternary weight network (TWN) [33] improved the binary quantization method by introducing 0 into the quantization values. These quantization methods allow CNNs to be deployed to FPGAs using a simple XNOR logic operation or additive and subtractive operations to implement convolution computation without occupying on-chip DSP resources at all, which not only significantly compresses the model size and saves FPGA computation and storage resources, but it also improves computational efficiency. However, they share the common drawback of excessive accuracy loss. Although recently developed methods, such as trained ternary quantization (TTQ) [34], learnable companding quantization (LCQ) [35], and smart quantization (SQ) [36], have significantly reduced quantization accuracy loss based on the traditional binary and ternary quantization methods, they are not completely hardware-friendly. As with many other methods, they do not quantize the first convolutional layer and the last fully connected layer in order to maintain the accuracy of quantized CNN models [20,21,33,34,37,38,39,40]. This leads to the need to continue using multiplication operations when deploying to FPGAs, and it is very difficult to implement these multiplication operations here because the unquantized layers are still 32-bit floating-point numbers, which require large on-chip computation resources and compromise computational efficiency.

However, incremental network quantization (INQ) [23] balances quantization accuracy and hardware adaptation capability better. INQ quantizes all the weights in a CNN model from a full-precision floating-point to powers of two or zero. It first proposed an iterative approach of three operations of weight partition, group-wise quantization, and retraining to make CNN models have a lower accuracy loss after all layers are quantized. In addition, weights in the form of powers of two make it possible to deploy CNNs on FPGA platforms using simple shift-logic operations instead of expensive multiplication operations without using DSP resources, resulting in a higher computational efficiency and a greater engineering significance. However, the INQ method still fails to achieve optimal quantization results at low-bit quantization. Some recent methods, such as MPA [41], also adopt the iterative approach of three operations of weight partition, group-wise quantization, and retraining proposed by INQ and introduce the clustering algorithm, etc., into the iterative to achieve higher quantization results at low-bit quantization. However, because MPA is not a power-of-two quantization method, it still requires a large number of multiplication operations when deployed to FPGAs, and the hardware-adaptation capability is limited.

In addition, unlike traditional power-of-two quantization methods, Gudovskiy et al. [24] and Chen et al. [42] both proposed algorithms that quantize the weights in CNNs directly to two power-of-two summations. Li et al. [37] further proposed the additive power-of-two quantization (APoT) algorithm by quantizing the weights to sums of multiple powers of two, depending on different settings. These quantization methods provide a denser interval of quantization values, solve the problem of the rigid resolution in power-of-two quantization, and have smaller accuracy loss. However, when deploying to FPGAs, the original single multiplication operation needs to be replaced with multiple shift operations and multiple addition operations without using on-chip DSPs and multiplication operations, because each weight is quantized as a multiple power-of-two summation. Compared with traditional power-of-two quantization methods, the consumed computational and storage resources increase exponentially, and the computational efficiency also decreases.

To better balance the quantization accuracy and hardware-adaptation capabilities in current quantization methods, in this paper we focus on the software and hardware co-optimization design method, presenting research on the low-bit “lossless” quantization of CNN models and its hardware-adaptation capability based on traditional power-of-two quantization methods and FPGA platforms. We proposed a more effective generic power-of-two quantization method: global sign-based network quantization (GSNQ). While maintaining the quantization framework comprising weight partition, group-wise quantization, and retraining, GSNQ develops a new weight-quantization strategy and adopts a more effective fine-grained and multi-scale global retraining method for the CNN models, which can significantly reduce the accuracy loss of the CNN models in low-bit quantization and obtain low-bit models with high accuracy.

Cong et al. [43] analyzed and counted the computational complexity of CNNs, finding that more than 90% of the computational complexity arises from convolutional computation, and the key to CNN deployment is the processing of convolutional computation. Therefore, we used shift operations to replace multiplication operations according to the characteristics of the power-of-two quantization, and we designed a novel convolutional computation module on FPGA platforms for GSNQ quantized models. This module does not occupy on-chip DSP resources and consumes less storage and fewer computational resources. Through this design, we verified that the GSNQ method has strong hardware-adaptation capability.

In summary, this study provides a reference for the industrial application of CNNs from both theoretical and experimental perspectives. The main work of this study is as follows:

1. We developed a sign-based power-of-two quantization strategy. The quantization value range was reasonably extended and the corresponding quantization rules were formulated according to the signs of weights. This strategy can prevent quantization loss caused by differences in the distribution of positive and negative weights, can ensure a wider quantized value range for each layer in the network, and can reduce the accuracy loss in low-bit quantization.
2. We proposed a fine-grained and multi-scale global retraining method, which adds vertical-level (inter-layer) iterations to horizontal-level (intra-layer) retraining in the original INQ method. The accuracy loss due to the change in parameters in one layer can be compensated by retraining all the other layers. This global retraining can compensate for the accuracy loss in low-bit quantization more effectively.
3. We reported on our performance of comparison quantization experiments on three classification datasets, MNIST [44], CIFAR10 [45], and Mini-ImageNet [46], each tested for LeNet-5 [44], AlexNet [2], VGG-16 [1], ResNet-18, ResNet-20, ResNet-56 [3], and GoogLeNet [47], to compare the proposed GSNQ method with INQ. We also compared the quantization results for the CIFAR10 dataset with other state-of-the-art quantization methods to comprehensively validate the effectiveness and generality of GSNQ.
4. We designed a high-performance convolutional computation module using shift operations instead of multiplication operations to complete the deployment of GSNQ quantized models on FPGA platforms. This module only uses abundant hardware resources, such as LUTs, to complete convolutional computation without consuming DSP resources. We compared this module with other mainstream multiplication-based convolutional computation modules to verify and analyze the hardware adaptability and engineering-application value of GSNQ.

The remaining parts of the paper are organized as follows. Section 2 describes the proposed method. Section 3 analyzes the details and results of the experiments. Finally, Section 4 concludes the study.

## 2. Proposed Method

### 2.1. Sign-Based Weight Quantization Strategy

GSNQ uses a quantization framework comprising weight partition, group-wise quantization, and retraining. For the weight-partition stage, it is widely believed that the larger the absolute value of the weights, the greater the impact on the results, according to previous experience and experiments [18,23,48]. In this study, we maintained this view, dividing the weights into different groups according to their absolute values, and then quantizing them group by group.

**Figure 1:**
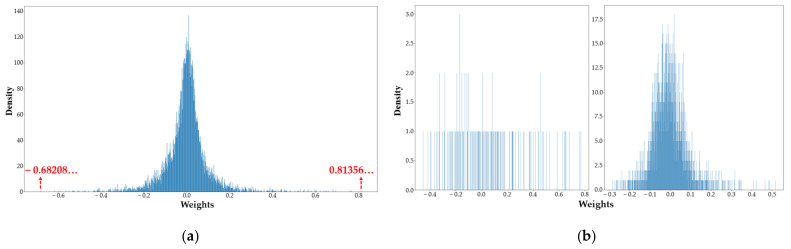
Weight distributions of CNNs. (**a**) Weight distribution of the first convolutional layer in GoogLeNet on Mini-ImageNet. (**b**) Weight distributions of the first and second convolutional layers in LeNet-5 on MNIST.

For the group-wise quantization stage, the quantized value range of INQ $P_{l}=\left\{{\pm \;2}^{n_{1}}{,\;\ldots ,\;\pm \;2}^{n_{2}},\;0\right\}$ is a symmetric set centered on 0, formulated based on the absolute values of weights. This range applies to the case in which the distribution of positive and negative weights in each layer of the CNN is relatively symmetric and the maximum absolute values of positive and negative weights are in an identical range. Figure 1 lists the weight distributions of some layers in some CNN models, where the horizontal coordinate indicates the range of weights and the vertical coordinate indicates the density of weights in that range.

It can be seen from Figure 1 that the macroscopic distribution of single-layer weights in CNNs generally obeys bell-shaped and long-tailed distributions [49]. However, in fact, there is no established rule for the specific weight distributions. In some layers, the distribution of positive and negative weight values in the long-tailed part has large variability, and the maximum absolute positive and negative weight values are not in the same range, as shown in Figure 1ab; therefore, the selection of the quantized value range in INQ is not universal. Meanwhile, the quantized value range boundaries in INQ are determined by the formula $n_{2}{=n}_{1}+1\;-{\;2}^{b-2}$, where ${\;n}_{1}$ and $n_{2}$ indicate the two bounds of the quantization value range in INQ, and b indicates the bit-width to which we want to quantize the weights. This approach does not fully utilize the expressiveness of the quantization bit-width binary numbers, and the quantization value range is set to a small extent. This suggests that INQ does not achieve the best quantization accuracy for this quantization bit-width. To address these issues, a sign-based weight-quantization strategy is proposed in this paper.

Suppose a pretrained full-precision (i.e., 32-bit floating-point) CNN model with a total of *L* layers can be represented by ${\{W}_{l\;}:\;1\;\leq \;l\;\leq \;L\}$. For the weight set $W_{l}$ of the l layer, we set a range of quantization values $P_{l}$:

(1) $$P_{l}=\left\{-2^{n_{4}}\;,\;\ldots \;,\;-2^{n_{3}}{\;,\;0\;,\;2}^{n_{2}}{\;,\;\ldots \;,\;2}^{n_{1}}\right\}$$

where $n_{1}$, $n_{2}$, $n_{3}$, and $n_{4}$ are four integer numbers, and $n_{4}\geq n_{3}$, $n_{1}\geq n_{2}$. We first need to determine $n_{1}$ and $n_{4}$, which are calculated using the following two equations:

(2) $$n_{1}{=\mathrm{floor}\;(\log}_{2}(\frac{{4s}_{1}}{3}))$$

(3) $$n_{4}{=\mathrm{floor}\;(\log}_{2}(\frac{{4s}_{2}}{3}))$$

where floor () indicates the round-down operation, and $s_{1}$ and $s_{2}$ indicate the absolute values of the maximum positive numbers and the minimum negative numbers in the weight set; they are, respectively, calculated by the following two formulas:

(4) $$s_{1}{=\max(W}_{l})$$

(5) $$s_{2}{=\mathrm{abs}(\min(W}_{l}))$$

where abs () means absolute value, max () means we take the maximum value of its input, and min () means we take the minimum value of its input. After determining $n_{1}$ and $n_{4}$, $n_{2}$ and $n_{3}$ can be calculated directly as:

(6) $$n_{2}{=n}_{1}-{\;2}^{b-1}+2$$

(7) $$n_{3}=n_{4}-2^{b-1}+2$$

where b indicates the bit-width to which we want to quantize the weights; it can be set directly according to requirements. We then obtain the full range of quantization values $P_{l}$.

Next, we start to quantize the weights of the grouped full-precision model into $P_{l}$ based on their signs. The quantization relationship for the positive weights $W_{l}\;(i)$ is as follows:

(8) $$W_{l}\;(i)=\left\{\begin{array}{l} \beta_{1}\;\;\;\;\;\;\;(\alpha_{1}+\beta_{1})/2\;\leq \;W_{l}(i)\;<3\beta_{1}/2\\ 0\;\;\;\;\;\;\;\mathrm{other}\;\mathrm{cases}\; \end{array}\right.$$

where $\alpha_{1}$ and $\beta_{1}$ are two adjacent elements in the range of positive quantization values $\left\{2^{n_{2}}{,\;\ldots ,\;2}^{n_{1}}\right\}$. Similarly, the quantization relationship for the negative weights $W_{l}\;(j)$ can be formulated as:

(9) $$W_{l}\;(j)=\left\{\begin{array}{l} \beta_{2}\;\;\;\;\;\;\;3\beta_{2}/2<W_{l}\;(j)\;\leq (\alpha_{2}+\beta_{2})/2\\ 0\;\;\;\;\;\;\;\mathrm{other}\;\mathrm{cases}\; \end{array}\right.$$

where $\alpha_{2}$ and $\beta_{2}$ are two adjacent elements in the range $\left\{-2^{n_{4}},\;\ldots ,\;-2^{n_{3}}\right\}$.

**Figure 2:**
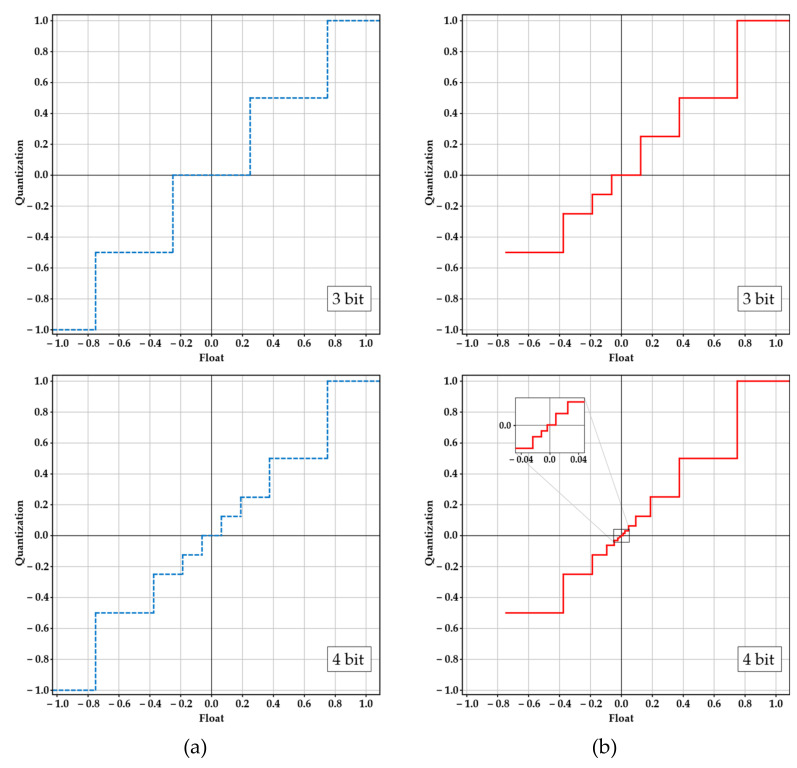
(**a**) The quantization strategies of INQ with different quantization bit-widths. (**b**) The quantization strategies of GSNQ with different quantization bit-widths.

Intuitively, the above weight quantization strategy is fundamentally based on determining the ranges of positive and negative quantization values using Equations (2), (3), (6) and (7); these two ranges can be the same or different, depending on the actual weight distributions. Figure 2 compares the quantization strategy of INQ with the GSNQ method for the convolution layer shown in Figure 1a.

The horizontal axis of Figure 2 represents the full-precision floating-point numbers before quantization, while the vertical axis of Figure 2 represents the quantization values. From Figure 2, it can be seen that the GSNQ method has more quantization values with the same quantization bit-width, which ensures the maximum range of quantization values. Furthermore, for this layer, GSNQ has different ranges of positive and negative quantization values. Taking 4-bit quantization as an example, GSNQ has a positive quantization value range $P_{\mathrm{lp}}=\left\{2^{-6},2^{-5},2^{-4},2^{-3},2^{-2},2^{-1},2^{0}\right\}$ and a negative quantization value range $P_{\ln\;}=\left\{-2^{-1},\;-2^{-2},\;-2^{-3},\;-2^{-4},\;-2^{-5},\;-2^{-6}{,\;2}^{-7}\right\},$ while $P_{\mathrm{lp}}=\left\{2^{-3}{,\;2}^{-2}{,\;2}^{-1}{,\;2}^{0}\right\}$ and $P_{\ln\;}=\left\{-2^{0},\;-2^{-1},\;-2^{-2},\;-2^{-3}\right\}$ in INQ. The negative minimum value in the weight distribution shown in Figure 1a is −0.68208; it is quantized as $-2^{-1}$, while no weight can be quantized as $-2^{0}$. Therefore, INQ has a useless quantization value in this case, $-2^{0}$, and there are only 7 actual quantization values for INQ. Therefore, it can be seen that the quantization strategy proposed in this paper extends the range of quantization values in all respects compared with the INQ method, which can reduce the accuracy loss caused by quantization more effectively and is also generally applicable to any weight distribution case in all CNNs.

### 2.2. Global Retraining Process

**Figure 3:**
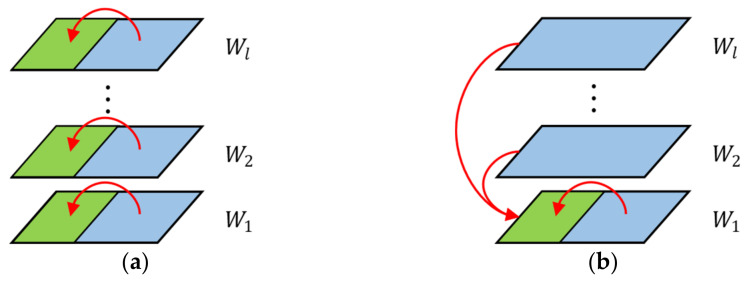
Comparison of two retraining approaches. (**a**) Horizontal retraining in INQ. (**b**) Global retraining in GSNQ. $W_{l}$ denotes the weight set of layer *l*, the green parts denote the quantized low-precision weight sets, the blue parts denote the full-precision weight sets used for retraining, and the arrows denote the loss-compensation process.

Each layer, and even each weight, plays an important role in the whole CNN model. Since the change in data caused by group-wise quantization causes accuracy loss in the original model, the model needs to be retrained to compensate for these losses. The retraining process in INQ is shown in Figure 3a, which is based on the horizontal level (intra-layers) of the network. Only the weights in the same layer are fine-tuned to compensate for the quantization loss of this layer. This approach only considers the linkage between parameters in a single layer and ignores the correlation between different layers in the training process, resulting in the inability to achieve the best results in quantization.

Therefore, we proposed a fine-grained and multi-scale global-based retraining approach in this paper, which iteratively retrains the CNN models at both horizontal (intra-layer) and vertical (inter-layer) levels, as shown in Figure 3b. Compared with the retraining process in INQ, the global retraining not only uses the weights in the same layer to compensate for the loss, but it also uses the remaining layers to compensate for the loss of one layer, which can reduce the accuracy loss of the quantized network more effectively.

**Figure 4:**
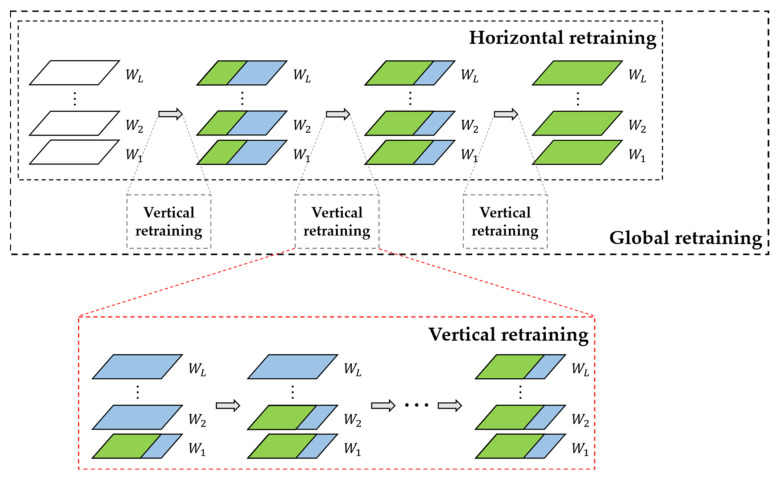
Global-based retraining process. $W_{l}$ denotes the weight set of layer *l*, the green areas denote the quantized low-precision weight sets, and the blue areas denote the full-precision weight sets used for retraining.

The complete flow chart of the proposed global retraining approach is shown in Figure 4. The partition and retraining approach in the INQ method is still used in this paper for the horizontal level. For the iterative process of the vertical level, we need to consider the importance of each layer in the CNN model to determine the iteration order. Han Song et al. [18] mentioned that, in the process of compression pruning, the first convolutional layer has a greater impact on the prediction accuracy compared to other convolutional layers with the same sparse ratio. Later, Tien-Ju Yang et al. [50] argued that the impact of the convolutional layer is greater than that of the fully connected layer. Based on these experiences and inferences, we developed a vertical-level iteration rule that iterates sequentially from the first to the last layer in CNN models.

First, we quantize the first group of weights in the first layer and use the global retraining method to compensate the quantization loss of this group. Next, the quantization and retraining of the first group of weights in all layers are completed by vertical-level iterations in turn. After quantizing the first group of weights in all layers, the next group of weights in the horizontal grouping of all layers is quantized and retrained by vertical-level iteration until all the weights are quantized.

In the global retraining process, the quantized weights must be kept fixed and not be involved in retraining. Therefore, we set a mask matrix $M_{l}$ to ensure that the weights in the weight-update stage during backpropagation are controllable. $M_{l}$ is defined as:

(10) $$M_{l}\;(a)=\left\{\begin{array}{l} 0\;\;\;\;\;\;W_{l}\;(a)\;\in Q_{l}\\ 1\;\;\;\;\;\;\mathrm{other}\;\mathrm{cases}\; \end{array}\right.$$

where $W_{l}\;(a)$ indicates the weights in layer *l* (no distinction between positive and negative), and $Q_{l}$ indicates the set of quantized weights in layer *l*.

Next, we use the stochastic gradient descent (SGD) algorithm to update the weights in the retraining process. The process is shown in Equation (11):

(11) $$W_{l}^{\prime }\;(a)\;\leftarrow W_{l}\;(a)\;-\;\gamma \frac{\partial E}{\partial (W_{l}\;(a))}M_{l}\left(a\right)$$

where γ indicates the learning rate, E indicates the loss function, $W_{l}\;(a)$ indicates the weight *a* in layer *l* (no distinction between positive and negative), and $W_{l}^{\prime }\;(a)$ represents the updated new weight. The formula shows that the weights are updated normally when $M_{l}(a)=1$ and are fixed when $M_{l}(a)=0$.

In summary, the algorithm of the proposed GSNQ method is shown in Algorithm 1.
**Algorithm 1:** Global Sign-Based Network Quantization.**Input:** Pretrained full-precision CNN model ${\{W}_{l\;}:\;1\;\leq \;l\;\leq \;l\}$**Output:** Quantized low-precision CNN model ${\{Q}_{l\;}:\;1\;\leq \;l\;\leq \;l\}$
1. Divide the weights of each layer into *N* groups ${\{D}_{n}^{\left(l\right)}:\;1\;\leq \;n\;\leq \;N\}$ according to the same predefined rules2:  **for** n ∈ [1, …, N] **do**3:    **for** l ∈ [1, …, l] **do**4:     Determine the quantization value range $P_{l\;}$ by Formulas (2), (3), (6) and (7)5:      Quantize weights in $D_{n}^{\left(l\right)}$ according to the weight sign by Formulas (8) and (9)6:      Retrain network and update weights by Formula (11)7:    **end for**8:  **end for**

### 2.3. Convolution Algorithm for GSNQ Based on FPGA

#### 2.3.1. Weight Recording

The 4-bit CNN models quantized by GSNQ have 15 quantization values per layer. Here, we need to recode these 15 numbers using 4-bit binary numbers and then store the encoded 4-bit binary numbers in storage units as new weights for transmission and calculation on FPGA platforms. Assuming that the range of quantization values is {−2^−1^, −2^−2^, −2^−3^, −2^−4^, −2^−5^, −2^−6^, −2^−7^, 0, 2^−6^, 2^−5^, 2^−4^, 2^−3^, 2^−2^, 2^−1^, 2^0^}, the recoding method is shown in Table 1:

The first bit of the 4-bit binary number is a sign bit, and the next three bits indicate the index value corresponding to the absolute value of that weight in Table 1. When performing the shift operation, the actual weights and numbers of shifted bits are determined based on these 4-bit numbers.

Similarly, there are 7 quantization values in each layer in the 3-bit quantized CNN models. Assuming that the quantization values range from {±2^−1^, ±2^−2^, ±2^−3^, 0}, the recoding method is shown in Table 2.

#### 2.3.2. Shift-Based Multiplication Processing Element

**Figure 5:**
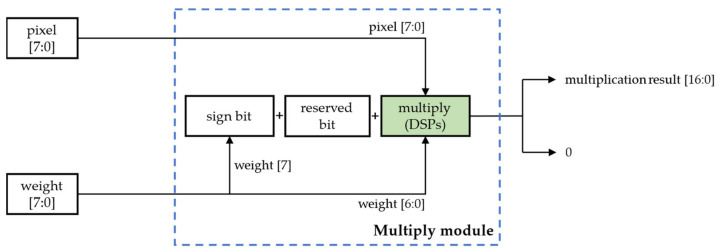
The multiplicative-based computation processing element.

The normal structure of the conventional multiplication-based computation processing element is shown in Figure 5 [15], where two input data values are fed directly to the on-chip DSP unit for multiplication operations.

**Figure 6:**
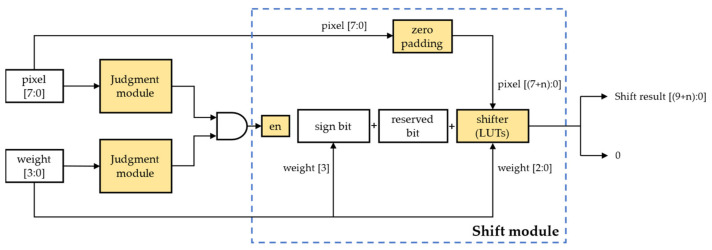
The shift-based multiplication processing element.

This method fundamentally limits the computational efficiency because of the enormous multiplication computation in CNNs and the availability of fewer DSP resources in the FPGA platforms. However, by quantizing the weights to powers of two, the complex multiplication operations can be replaced by simple shift-logic operations. The shift operation is mainly implemented by on-chip LUT logic units, and compared with on-chip DSPs, LUT resources on FPGAs are relatively abundant, with a higher processing efficiency and a lower energy consumption. Therefore, we took a low-bit GSNQ-quantized CNN model as an example and designed a novel shift-based multiplication processing element without DSP resources, which is shown in Figure 6.

There are two input data and one output datum in each shift-based PE; one input datum is a feature-map pixel (8-bit unsigned positive number), while the other is a weight (4-bit signed number). The output data can be a signed number or zero. A judgment module was added to judge whether there are any zero values in the input data. This module can output a low-level signal when the input data is 0 and a high-level signal when the input data is not 0. Then, these two signals are sent to the AND gate to generate an enable signal. The shift module starts to run only when the enable signal is high, otherwise it outputs a zero directly. Before starting the shift operation, the input feature map pixel needs to be padded with zeros and then input to the shifter together with the weight to obtain the shifted result; the specific number of zero padding depends on the weights in the convolutional layer. At the same time, we use the highest bit of the recoded weight as the sign bit of the output data, and we also set a reserved bit to prevent overflow during the subsequent data accumulation. These three parts form the output data of this PE together.

#### 2.3.3. Shift-Based Convolutional Computation Module

**Figure 7:**
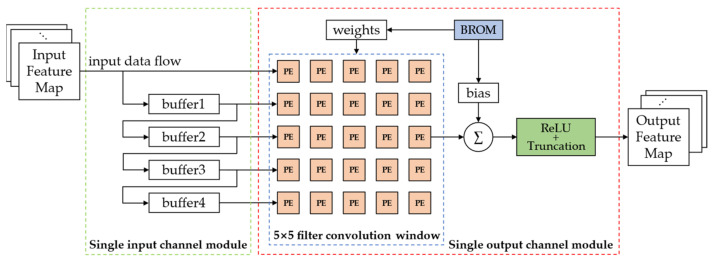
The shift-based convolutional computation module.

According to the PE in the previous section, we designed a shift-based convolutional computation module with a 5 × 5 convolutional kernel as an example, as shown in Figure 7.

**Figure 8:**
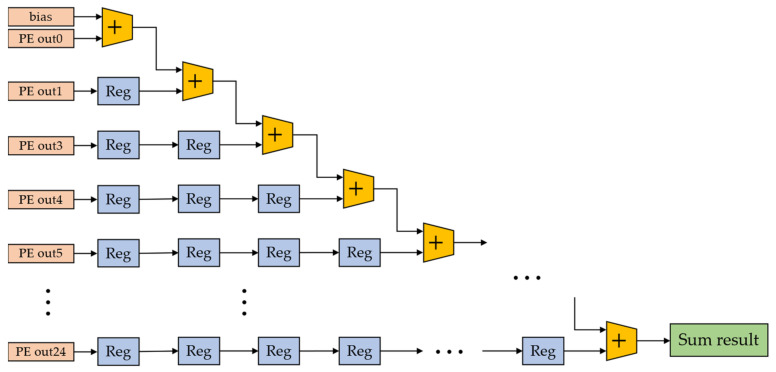
The designed accumulator.

This shift-based convolutional computation module consists of two main parts: a single input channel module and a single output channel module. In the single input channel module, 4 buffers (depending on the size of the convolution kernel) with a depth equal to the width of the input feature map were designed to reuse data. The feature map is input to the single input channel module as a data stream and stored in buffers in order. Then, the feature data are input to the single output channel module. This module was designed with a 5 × 5 array of shift-based computation processing elements, which forms a convolutional sliding window together with the feature-map pixels. Here, we store the CNN parameters without off-chip DDR and only on-chip ROM, because when the parameters are quantized as 4-bit, 3-bit, or 2-bit, most CNN models can be compressed within 10 Mb. This can improve the read rate and reduce energy consumption. The sliding window outputs 25 shift operation results per clock cycle. These 25 shift operation results are then accumulated with the corresponding bias by an accumulator to obtain a signed convolution result. The designed accumulator is shown in Figure 8.

In the accumulator, we designed 25 adders and arranged them in a pipelined manner. Each adder performs simple addition of binary numbers. The output of the sliding window moves from left to right in an order according to the clock cycle and is processed accordingly. Reg represents the register in which the output of the sliding window is temporarily stored when no addition operation is performed during the current clock cycle. The final result of the accumulator is output to the activation function and truncation module.

**Figure 9:**
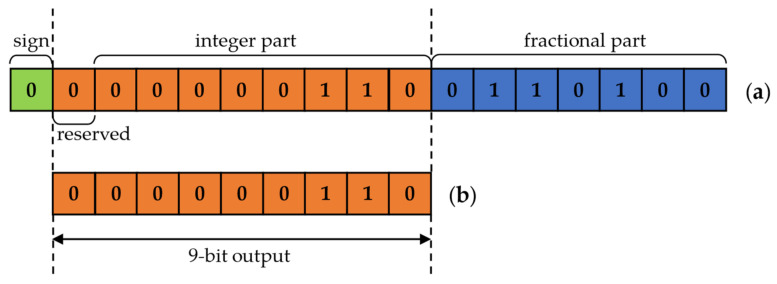
An example: (**a**) output data before truncation; (**b**) output data after truncation.

When passing through the ReLU activation-function module, negative results are directly output as 0, while positive results are truncated by the sign bit and the decimal part to output an unsigned number, as shown in Figure 9.

This truncation method not only ensures lower accuracy loss, but it also saves transmission bandwidth. All output data form an output feature map that is used as input to the next layer.

The design of the whole convolutional layer can set the parallelism of input and output channels according to the actual situation, such as the number of on-chip hardware resources and the required computation rate, and the parallel processing of the input and output channels can be achieved by using the corresponding number of single input channel modules and single output channel modules in Figure 7.

## 3. Experiments

In this section, we directly verify the effectiveness and generality of the proposed GSNQ method by describing quantization comparison experiments on the software side, and we indirectly verify the hardware adaptability and engineering application value of GSNQ by describing our research on the effectiveness of the designed shift-based convolutional algorithm on the hardware side.

### 3.1. GSNQ Quantitative Comparison Experiments

#### 3.1.1. Implementation Details

First, we quantized LeNet-5 on the MNIST dataset, and we quantized AlexNet, VGG-16, ResNet, and GoogLeNet on the CIFAR10 dataset and Mini-ImageNet dataset, respectively, to evaluate the effectiveness and generality of GSNQ.

We trained these original CNN models, provided by the PyTorch library [51], for 200 epochs on three datasets to obtain the 32-bit full-precision floating-point baseline models for quantization (the pretrained baseline models for ResNet-20 and ResNet-56 on CIFAR10 dataset are provided in [3]; we used them directly). For INQ, we followed the setup in [23], with 12 epochs of retraining for 4-bit quantization and 20 epochs of retraining for 3-bit quantization. For GSNQ, we set 7 epochs of retraining for both 4-bit and 3-bit quantization and 15 epochs for 2-bit quantization. Other important training parameters are shown in Table 3; they were kept consistent when performing the ablation experiments for comparison.

Our software experiments were built based on the PyTorch library, and the development environment was Pycharm Community Edition 2021.2.3. We also reproduced the INQ algorithm based on the PyTorch library according to the description in [23]. The training platform was an NVIDIA GeForce RTX 2080Ti GPU.

#### 3.1.2. Ablation Experiments

We conducted ablation experiments on the above CNNs for three datasets to perform a comprehensive analysis of GSNQ and to verify its effectiveness and generality. The experimental results are shown in Table 4, Table 5, Table 6, Table 7 and Table 8. Table 4 compares the 4-bit and 3-bit quantization results of LeNet-5 on the MNIST dataset. Table 5 and Table 6 show the quantization results of the CIFAR10 dataset, and Table 7 and Table 8 show the quantization results of the Mini-ImageNet dataset. “GSNQ (only S)” denotes the GSNQ method that only changes the weight quantization strategy without global retraining, and “GSNQ (only G)” denotes the GSNQ method that does not change the weight quantization strategy, but it only adds a global retraining method. The numbers in red for these tables indicate the results of our approach.

It can be observed from the results of the ablation experiments that the INQ method had good results in 4-bit quantization, and there are cases in which the model accuracy was slightly lower or higher than that of the full-precision baseline model, which basically achieves lossless quantization. Only improving the weight quantization strategy (i.e., GSNQ (only S)) and only adding global retraining (i.e., GSNQ (only G)) both achieve a completely lossless quantization; the quantization results were all higher than those of the full-precision baseline models and INQ quantized models. In addition, the results can be said to prove that, if INQ can achieve almost lossless quantization, only improving the weight-quantization strategy has little effect on the results, while adding global retraining can compensate for the loss more effectively and improve the accuracy of the quantized models to a greater extent. GSNQ combines the two improvements, and it can be seen that the quantization results from all the CNN models greatly exceeded the full-precision baseline models. The Top-1 accuracy generally increased by about 2–3%, with optimal results. Even for AlexNet and VGG-16 on the Mini-ImageNet dataset, the Top-1 and Top-5 accuracies of the 4-bit GSNQ quantized models were 7.14%/3.84% and 5.28%/1.86%, which were higher, surprisingly, than the baseline models.

However, the INQ began to struggle in 3-bit quantization, with the accuracy of some CNN models dropping significantly. For example, the Top-1 accuracy of AlexNet and VGG-16 on the CIFAR10 dataset decreased by 15.41% and 9.00%, respectively, and that of GoogLeNet on the Mini-ImageNet dataset decreased by 9.59%. Changing the weight-quantization strategy (GSNQ (only S)) can greatly reduce the accuracy loss, making 3-bit quantization close to a lossless quantization again. This is because the useless quantization values of INQ in 3-bit quantization due to sign problems cause the very small quantization value range to become even smaller, and the proposed weight-quantization strategy not only solves this problem, but it also reasonably and effectively expands the range of quantization values. In addition, when the INQ has huge accuracy loss, only increasing global retraining cannot achieve the effect of the GSNQ (only S), but it can only reduce the quantization loss to a limited extent on the basis of INQ, and it still cannot achieve lossless quantization. For example, the Top-1 accuracy of AlexNet on the CIFAR10 dataset after GSNQ (only G) quantization still decreased by 7.11%, while it only decreased by 2.37% after GSNQ (only S) quantization. Finally, GSNQ combined the two improvements and also achieved a completely lossless quantization of all the CNN models at 3-bit quantization. The Top-1 accuracy of AlexNet on the Mini-ImageNet dataset even rose by 6.06% compared to the baseline model. Further experiments also demonstrated that, for the 4-bit and 3-bit quantization of GSNQ, seven epochs per retraining can achieve the optimal results, and more epochs have little effect on the final quantization results.

In conclusion, multiple groups of ablation experiments demonstrated the effectiveness of the two improvements in GSNQ. The new weight-quantization strategy fundamentally solves the problem of excessive accuracy loss, while global retraining can further improve the accuracy of the quantized model on this basis. The two improvements complement each other and can make the quantized low-bit fixed-point models have a higher accuracy than the full-precision models. The experimental results also prove that the GSNQ method has strong generality and is suitable for different CNNs and different weight distributions.

#### 3.1.3. Comparison with State-of-the-Art Quantization Methods

To further validate the proposed GSNQ method, we compared GSNQ with some state-of-the-art quantization methods and evaluated them comprehensively. We quantized the baseline CNN models trained on the CIFAR10 dataset to 4-bit and 3-bit by the codes provided in [37,38], and the results obtained are shown in Table 9.

It can be observed in Table 9 that the Top-1 accuracies of the three methods were all higher than those of the baseline models. The GSNQ method had the highest accuracy in 4-bit quantization.

In addition, many past works quantized the ResNet-20 and ResNet-56 models on the CIFAR10 dataset, and paper [3] provides the pretrained models for them. In this paper, we used GSNQ to quantize the provided baseline models directly with the parameters set, as shown in Table 3. The quantization results were compared, as shown in Table 10, where the results of DoReFa-Net [52], PACT [40], LQ-Net [39], ProxQuant [53], and APoT [37] were taken from their original papers.

It can be seen that the accuracies of the 4-bit and 3-bit models quantized by GSNQ completely surpassed those of the full-precision baseline models. The accuracy of the 4-bit GSNQ quantized model of ResNet-20 was higher than that of all other methods, and the accuracy of the 4-bit GSNQ quantized model of ResNet-56 was on par with the APoT model. The 2-bit GSNQ quantized models did not reach the accuracy of the baseline models. However, the accuracy loss was small, which still surpassed most existing quantization methods.

The accuracy of GSNQ was slightly lower than APoT in 3-bit quantization. However, because the APoT method actually quantizes weights to sums of multiple powers of two, it requires additional shift and addition operations compared to the GSNQ method when deployed to FPGA platforms, which makes the storage and computational resource occupation in hardware increase exponentially; the computational efficiency is also affected. Therefore, combining the performance of both software and hardware, we can confirm that the GSNQ method has better prospects for engineering applications.

### 3.2. FPGA Design Experiments

#### 3.2.1. Implementation Details

We compared the designed high-performance convolutional computation module based on shift operations with other methods based on multiplication operations on an FPGA platform to verify the hardware-adaptation capability of GSNQ. The size of the input feature map used in the experiments was 32 × 32 pixels. The entire FPGA experiment was conducted based on a MSXBO Zynq-7000 MZ7035FA development board with a ZYNQ XC7Z035FFG676-2I chip on board, which contains 171,900 LUTs, 343,800 FFs, and 900 DSPs. The FPGA engineering code was written in Verilog hardware description language, and the development environment was Vivado Design Suite, HLx Editions 2019.2, provided by Xilinx.

#### 3.2.2. Results Analysis

We compared our designed module with two other conventional multiplication-based convolutional computation modules: Method 1, which implements multiplication operations using on-chip DSPs [15], and Method 2, which implements multiplication operations using on-chip LUTs by the hardware-synthesis function in the Vivado Design Suite [54]. Table 11 shows the on-chip hardware-resource occupation comparison of these three convolutional computation modules.

It can be observed in Table 11 that the traditional convolutional computation module using on-chip DSPs for multiplication operations consumes a large number of on-chip DSPs regardless of the bit width of weights. Using the Vivado Design Suite to implement multiplication operations directly with on-chip LUTs can be completely free of the on-chip DSPs. However, as a price, the occupation of LUTs increases significantly, even by more than 62% at 3 bits. Moreover, the shift-based convolutional computation module designed in this paper also does not occupy DSPs at all. Additionally, compared with Method 1, the resource occupation of LUTs increases slightly, and the resource occupation of FFs decreases. This is because the shift operation is implemented by the LUTs. Compared with Method 2, the resource occupation of the LUTs in our design is significantly reduced without using DSP resources as well.

Furthermore, since the number of input and output channels of the convolutional layer in a convolutional neural network is usually a multiple of 32, general FPGA deployment involves setting up a computing module with 32 × 32 input and output channel parallelism as a basic processing unit for convolutional computation [55,56,57,58] and then considering whether to continue to stack this basic unit according to the actual situation. Therefore, we also set up a 32-way parallel processing convolutional computation basic processing unit to compare the three methods to verify the performance of our method when completing basic parallel convolutional computation, and Table 12 shows the hardware-resource occupation of our method compared with the other two methods at 32-channel parallelism.

It can be observed in Table 12 that the on-chip DSP occupancy in Method 1 is close to 90%. If the basic processing unit is stacked again, the DSP resources on the XC7Z035 chip will become insufficient, and it is necessary to use an FPGA chip with more on-chip resources, which also undoubtedly leads to an increase in costs. However, our approach does not occupy the on-chip DSP resources, and it also has a lower combined occupancy of other on-chip hardware resources. Meanwhile, without the limitation of DSP resources, we can continue to stack this 32-channel parallelism basic processing unit upwards. It is obvious that the shift-based convolutional computation algorithm proposed in this paper achieves overall savings of on-chip resources on FPGA platforms, which can result in greater parallelism to obtain higher computational efficiency. This is proof that GSNQ has strong hardware-adaptation capability and engineering-application value.

## 4. Conclusions

In this paper, we proposed an innovative, hardware-friendly, low-bit quantization method: global sign-based network quantization (GSNQ). The GSNQ method adopts the quantization framework of weight partition, group-wise quantization, and retraining. In the group-wise quantization stage, GSNQ sets the different quantization rules according to the sign of the weights in each layer in the full-precision CNN models and provides the largest quantization-value range. Furthermore, in the retraining stage, GSNQ introduces a fine-grained and multi-scale global retraining operation, which can compensate for the accuracy loss caused by low-bit quantization to the greatest extent, so that the accuracy of the quantized models can maintain or even surpass that of the original full-precision models. In addition, GSNQ quantizes the weights to powers of two or zero. According to this feature, we designed a novel convolutional algorithm based on shift operation, which achieved 0 DSP occupancy. The results of the quantization comparison experiments show that GSNQ achieves low-bit “lossless” quantization and obtains quantization results at an advanced level for different models and different datasets. The results of the FPGA deployment experiments show that our convolutional algorithm achieves the least on-chip hardware resource occupation, proving that GSNQ has strong hardware-adaptation capability, which facilitates the improvement of computational parallelism and efficiency. Our research on the software and hardware co-optimization design of CNNs provides theoretical and experimental support for the industrial application of CNNs, which can realize a CNN accelerator with excellent comprehensive performance.

## Figures and Tables

**Table 1:** Recoding weights (4-bit).

Recoded Weight	Quantized Weight	Recoded Weight	Quantized Weight
0001	2^−1^	1001	−2^−1^
0010	2^−2^	1010	−2^−2^
0011	2^−3^	1011	−2^−3^
0100	2^−4^	1100	−2^−4^
0101	2^−5^	1101	−2^−5^
0110	2^−6^	1110	−2^−6^
0111	2^0^	1111	−2^−7^
0000	0	Null	Null

**Table 2:** Recoding weights (3-bit).

Recoded Weight	Quantized Weight	Recoded Weight	Quantized Weight
001	2^−1^	101	−2^−1^
010	2^−2^	110	−2^−2^
011	2^−3^	111	−2^−3^
000	0	Null	Null

**Table 3:** Training parameters for different CNNs.

Network	Weight Partition	Weight Decay	Momentum	Learning Rate	Batch Size
LeNet-5	(0.3, 0.6, 0.8, 1)	0.0001	0.9	0.1	256
AlexNet	(0.3, 0.6, 0.8, 1)	0.0005	0.9	0.01	256
VGG-16	(0.5, 0.75, 0.875, 1)	0.0005	0.9	0.01	128
ResNet-18	(0.5, 0.75, 0.875, 1)	0.0005	0.9	0.01	128
ResNet-20	(0.2, 0.4, 0.6, 0.8, 1)	0.0001	0.9	0.1	256
ResNet-56	(0.2, 0.4, 0.6, 0.8, 1)	0.0001	0.9	0.1	128
GoogLeNet	(0.2, 0.4, 0.6, 0.8, 1)	0.0002	0.9	0.01	128

**Table 4:** Ablation experiments on LeNet-5 on the MNIST dataset.

Network	Method	Bit Width	Top-1 Accuracy	Top-5 Accuracy	Increase in Top-1/Top-5 Error
LeNet-5	Baseline	32-bit	98.84%	100.00%	
	INQ	4-bit	97.86%	99.97%	−0.98%/−0.03%
	GSNQ (only S)	4-bit	98.01%	99.97%	−0.83%/−0.03%
	GSNQ (only G)	4-bit	98.48%	99.99%	−0.36%/−0.01%
	GSNQ	4-bit	98.86%	100.00%	0.02%/0.00%
	INQ	3-bit	96.11%	99.84%	−2.73%/−0.16%
	GSNQ (only S)	3-bit	97.67%	99.97%	−1.17%/−0.03%
	GSNQ (only G)	3-bit	97.01%	99.88%	−1.83%/−0.12%
	GSNQ	3-bit	98.33%	99.99%	−0.51%/−0.01%

**Table 5:** Ablation experiments on 4-bit quantization on the CIFAR10 dataset.

Network	Method	Bit Width	Top-1 Accuracy	Top-5 Accuracy	Increase in Top-1/Top-5 Error
AlexNet	Baseline	32-bit	81.26%	98.90%	
	INQ	4-bit	79.92%	98.64%	−1.34%/−0.26%
	GSNQ (only S)	4-bit	81.61%	99.07%	0.35%/0.17%
	GSNQ (only G)	4-bit	82.83%	99.11%	1.57%/0.21%
	GSNQ	4-bit	84.62%	99.26%	3.36%/0.36%
VGG-16	Baseline	32-bit	85.33%	99.39%	
	INQ	4-bit	85.94%	99.49%	0.61%/0.10%
	GSNQ (only S)	4-bit	86.17%	99.51%	0.84%/0.12%
	GSNQ (only G)	4-bit	87.92%	99.55%	2.59%/0.16%
	GSNQ	4-bit	88.12%	99.59%	2.79%/0.20%
ResNet-18	Baseline	32-bit	88.88%	99.65%	
	INQ	4-bit	89.18%	99.66%	0.30%/0.01%
	GSNQ (only S)	4-bit	89.20%	99.68%	0.32%/0.03%
	GSNQ (only G)	4-bit	90.09%	99.65%	1.21%/0.00%
	GSNQ	4-bit	90.46%	99.75%	1.58%/0.10%
GoogLeNet	Baseline	32-bit	89.00%	99.60%	
	INQ	4-bit	89.37%	99.62%	0.37%/0.02%
	GSNQ (only S)	4-bit	89.49%	99.61%	0.49%/0.01%
	GSNQ (only G)	4-bit	90.46%	99.66%	1.46%/0.06%
	GSNQ	4-bit	90.72%	99.66%	1.72%/0.06%

**Table 6:** Ablation experiments on 3-bit quantization on the CIFAR10 dataset.

Network	Method	Bit Width	Top-1 Accuracy	Top-5 Accuracy	Increase in Top-1/Top-5 Error
AlexNet	Baseline	32-bit	81.26%	98.90%	
	INQ	3-bit	65.85%	95.95%	−15.41%/−2.95%
	GSNQ (only S)	3-bit	78.89%	98.73%	−2.37%/−0.17%
	GSNQ (only G)	3-bit	74.15%	98.18%	−7.11%/−0.72%
	GSNQ	3-bit	82.14%	99.09%	0.88%/0.19%
VGG-16	Baseline	32-bit	85.33%	99.39%	
	INQ	3-bit	76.33%	98.50%	−9.00%/−0.89%
	GSNQ (only S)	3-bit	85.18%	99.44%	−0.15%/0.05%
	GSNQ (only G)	3-bit	77.47%	98.53%	−7.86%/−0.86%
	GSNQ	3-bit	87.59%	99.58%	2.26%/0.19%
ResNet-18	Baseline	32-bit	88.88%	99.65%	
	INQ	3-bit	85.56%	99.58%	−3.32%/−0.07%
	GSNQ (only S)	3-bit	88.84%	99.69%	−0.04%/0.04%
	GSNQ (only G)	3-bit	87.64%	99.61%	−1.24%/−0.04%
	GSNQ	3-bit	90.01%	99.58%	1.13%/−0.07%
GoogLeNet	Baseline	32-bit	89.00%	99.60%	
	INQ	3-bit	86.08%	99.38%	−2.92%/−0.22%
	GSNQ (only S)	3-bit	89.07%	99.63%	−0.07%/0.03%
	GSNQ (only G)	3-bit	89.85%	99.53%	0.85%/−0.07%
	GSNQ	3-bit	90.70%	99.60%	1.70%/0.00%

**Table 7:** Ablation experiments on 4-bit quantization on the Mini-ImageNet dataset.

Network	Method	Bit Width	Top-1 Accuracy	Top-5 Accuracy	Increase in Top-1/Top-5 Error
AlexNet	Baseline	32-bit	58.05%	83.50%	
	INQ	4-bit	58.33%	83.43%	0.28%/−0.07%
	GSNQ (only S)	4-bit	59.31%	83.98%	1.26%/0.48%
	GSNQ (only G)	4-bit	64.27%	86.82%	6.22%/3.32%
	GSNQ	4-bit	65.19%	87.43%	7.14%/3.84%
VGG-16	Baseline	32-bit	73.68%	91.03%	
	INQ	4-bit	73.48%	90.94%	−0.20%/−0.09%
	GSNQ (only S)	4-bit	73.91%	91.06%	0.23%/0.03%
	GSNQ (only G)	4-bit	78.33%	92.70%	4.65%/1.67%
	GSNQ	4-bit	78.96%	92.89%	5.28%/1.86%
ResNet-18	Baseline	32-bit	74.43%	92.11%	
	INQ	4-bit	74.37%	91.96%	−0.06%/−0.15%
	GSNQ (only S)	4-bit	74.83%	91.96%	0.40%/−0.15%
	GSNQ (only G)	4-bit	78.30%	92.92%	3.87%/0.81%
	GSNQ	4-bit	78.49%	92.92%	4.06%/0.81%
GoogLeNet	Baseline	32-bit	77.76%	93.40%	
	INQ	4-bit	77.83%	93.42%	0.07%/0.02%
	GSNQ (only S)	4-bit	78.37%	93.56%	0.61%/0.16%
	GSNQ (only G)	4-bit	79.49%	93.77%	1.73%/0.37%
	GSNQ	4-bit	79.96%	93.52%	2.20%/0.12%

**Table 8:** Ablation experiments on 3-bit quantization on the Mini-ImageNet dataset.

Network	Method	Bit Width	Top-1 Accuracy	Top-5 Accuracy	Increase in Top-1/Top-5 Error
AlexNet	Baseline	32-bit	58.05%	83.50%	
	INQ	3-bit	49.48%	77.26%	−8.57%/−6.24%
	GSNQ (only S)	3-bit	57.29%	83.29%	−0.76%/−0.21%
	GSNQ (only G)	3-bit	52.87%	81.26%	−5.18%/−2.24%
	GSNQ	3-bit	64.11%	86.70%	6.06%/3.20%
VGG-16	Baseline	32-bit	73.68%	91.03%	
	INQ	3-bit	71.27%	90.17%	−2.14%/−0.86%
	GSNQ (only S)	3-bit	73.60%	91.04%	−0.08%/0.01%
	GSNQ (only G)	3-bit	74.44%	91.45%	0.76%/0.42%
	GSNQ	3-bit	75.69%	92.10%	2.01%/1.07%
ResNet-18	Baseline	32-bit	74.43%	92.11%	
	INQ	3-bit	69.81%	89.62%	−4.62%/−2.49%
	GSNQ (only S)	3-bit	73.82%	91.38%	−0.61%/−0.73%
	GSNQ (only G)	3-bit	76.51%	92.26%	2.08%/0.15%
	GSNQ	3-bit	77.69%	92.86%	3.26%/0.75%
GoogLeNet	Baseline	32-bit	77.29%	93.14%	
	INQ	3-bit	67.70%	88.95%	−9.59%/−4.19%
	GSNQ (only S)	3-bit	76.98%	93.12%	−0.31%/−0.02%
	GSNQ (only G)	3-bit	74.76%	91.57%	−2.53%/−1.57%
	GSNQ	3-bit	79.24%	93.49%	1.95%/0.35%

**Table 9:** Comparison with the state-of-the-art quantization methods on CIFAR10.

Network	Method	Bit Width	Top-1 Accuracy	Top-5 Accuracy	Increase in Top-1/Top-5 Error
ResNet-18	Baseline	32-bit	88.88%	99.65%	
	DSQ	4-bit	90.38%	99.66%	1.50%/0.01%
	APoT	4-bit	90.20%	99.69%	1.32%/0.04%
	GSNQ	4-bit	90.46%	99.75%	1.58%/0.10%
	DSQ	3-bit	90.01%	99.68%	1.13%/0.03%
	APoT	3-bit	90.18%	99.58%	1.30%/−0.07%
	GSNQ	3-bit	90.01%	99.58%	1.13%/−0.07%
GoogLeNet	Baseline	32-bit	89.00%	99.60%	
	DSQ	4-bit	90.47%	99.63%	1.47%/0.03%
	APoT	4-bit	89.91%	99.49%	0.91%/−0.11%
	GSNQ	4-bit	90.72%	99.66%	1.72%/0.06%
	DSQ	3-bit	89.99%	99.57%	0.99%/−0.03%
	APoT	3-bit	90.78%	99.61%	1.78%/0.01%
	GSNQ	3-bit	90.70%	99.60%	1.70%/0.00%

**Table 10:** Comparison between ResNet-20 and ResNet-56 on CIFAR10.

Network	Method	Top-1 Accuracy		
		4-bit	3-bit	2-bit
ResNet-20 (Baseline: 91.60%)	DoReFa-Net	90.5%	89.9%	88.2%
	PACT	91.7%	91.1%	89.7%
	LQ-Net	—	91.6%	90.2%
	ProxQuant	—	—	90.6%
	APoT	92.3%	92.2%	91.0%
	GSNQ	92.42%	91.96%	90.91%
ResNet-56 (Baseline: 93.20%)	APoT	94.0%	93.9%	92.9%
	GSNQ	94.00%	93.62%	92.92%

**Table 11:** On-chip resource occupation of a single convolution computation module based on different methods.

Method	Bit Width	LUTs	FFs	DSPs
Method 1: Using on-chip DSPs	4-bit	1036	790	25
Method 2: Using on-chip LUTs	4-bit	1373	690	0
Our method	4-bit	1094	734	0
Method 1: Using on-chip DSPs	3-bit	680	691	25
Method 2: Using on-chip LUTs	3-bit	1103	585	0
Our method	3-bit	722	612	0

**Table 12:** On-chip resource occupation for convolutional computation at 32-channel parallelism.

Method	Bit Width	LUTs	FFs	DSPs
Method 1: Using on-chip DSPs	4-bit	38,356 (22.31%)	28,171 (8.19%)	800 (88.89%)
Method 2: Using on-chip LUTs	4-bit	54,383 (31.64%)	24,971 (7.26%)	0 (0.00%)
Our method	4-bit	41,923 (24.39%)	28,151 (8.19%)	0 (0.00%)
Method 1: Using on-chip DSPs	3-bit	23,303 (13.56%)	24,968 (7.26%)	800 (88.89%)
Method 2: Using on-chip LUTs	3-bit	37,719 (21.94%)	22,568 (6.56%)	0 (0.00%)
Our method	3-bit	30,163 (17.55%)	23,330 (6.79%)	0 (0.00%)

## Data Availability

Not applicable.
